# Multispectral Laser‐Scanning Photoacoustic Microscopy With SRS‐Generated Wavelengths for Skin Chromophore Characterization

**DOI:** 10.1002/jbio.70225

**Published:** 2026-01-23

**Authors:** Amir Khansari, Seyed Mohsen Ranjbaran, Mohsin Zafar, Nafiseh Ghaffar Nia, Hossein Khodavirdi, Maria Tsoukas, Kamran Avanaki

**Affiliations:** ^1^ The Richard and Loan Hill Department of Biomedical Engineering University of Illinois at Chicago Chicago USA; ^2^ Department of Dermatology and Paediatrics University of Illinois at Chicago Chicago USA

**Keywords:** cross‐correlation, deep learning, multispectral photoacoustic microscopy, photoacoustic, signal decomposition

## Abstract

We present a single pump‐source, multispectral laser‐scanning photoacoustic microscopy (MS‐LS‐PAM) platform that integrates stimulated Raman scattering (SRS)‐based wavelength generation for high‐resolution, label‐free skin imaging. The system uses a compact, fiber‐based light source to produce tunable excitation wavelengths from 532 to 571 nm, enabling precise spectral discrimination of key endogenous chromophores, oxyhemoglobin (HbO_2_), deoxyhemoglobin (Hb), melanin, and collagen, without external contrast agents. Spectral unmixing is performed using non‐negative least squares (NNLS), facilitating quantitative analysis of chromophore distributions. In vivo experiments on B6 (pigmented) and SKH1 hairless (melanin deficient) mice demonstrated accurate chromophore separation, spatially resolved oxygen saturation (sO_2_) mapping, and clear visualization of collagen and melanin architectures.

## Graphical abstract

Unmixed maps showing vascular oxygen saturation (sO_2_), relative collagen, and relative melanin distributions. Maps were extracted from multispectral imaging of B6 and SKH1 Nude mouse abdominal skin using the proposed MS‐LS‐PAM system.

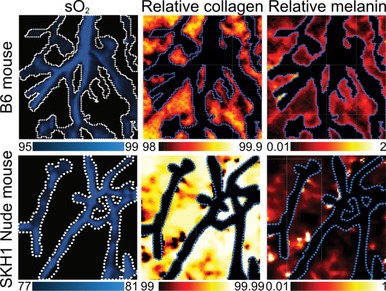

## 1 Introduction

Photoacoustic microscopy (PAM) is a hybrid biomedical imaging modality that combines optical excitation with ultrasonic detection to generate high‐resolution, high‐contrast images based on the optical absorption of biological tissues [1]. In PAM, pulsed laser light is absorbed by endogenous chromophores such as hemoglobin and melanin, producing thermoelastic expansion and the emission of ultrasonic waves, which are detected to form images. Optical‐resolution PAM (OR‐PAM), which achieves micrometer‐scale lateral resolution through tight optical focusing, is particularly suited for superficial tissue visualization, including microvasculature and skin pigmentation [2]. Laser‐scanning PAM (LS‐PAM) further enhances imaging speed and system simplicity by raster‐scanning the laser beam while keeping the ultrasonic transducer stationary, enabling fast, high‐fidelity imaging of large areas without mechanical transducer movement [3]. To extend PAM beyond structural imaging, multispectral PAM (MS‐PAM) acquires data at multiple optical wavelengths to exploit the distinct absorption spectra of chromophores such as oxyhemoglobin (HbO_2_), deoxyhemoglobin (Hb), melanin, and collagen [4]. This capability supports functional imaging tasks such as mapping blood oxygen saturation (sO_2_), quantifying melanin and collagen content, and assessing tissue composition, critical for dermatological diagnostics. In dermatologic imaging, a wide spectrum of noninvasive modalities is already used to aid melanoma and non‐melanoma skin cancer diagnosis. FDA‐ and EMA‐approved devices based on high‐frequency ultrasound, reflectance confocal microscopy, and several OCT implementations are now being evaluated for routine basal cell carcinoma subtyping and treatment planning [5]. Recent reviews have summarized the performance of these imaging technologies in cutaneous melanoma and positioned photoacoustic imaging among emerging tools for assessment and staging [2, 6]. Complementary work on melanoma biomarkers has highlighted pigment, vascular morphology, and tumor‐microenvironment signatures as promising targets for in vivo imaging [7]. Early OCT‐based computer‐aided diagnosis studies further showed that optical scattering signatures and image‐derived features can differentiate basal cell carcinoma from normal skin [8]. Within this landscape, multispectral photoacoustic microscopy offers complementary absorption‐based contrast with intrinsic sensitivity to hemoglobin oxygenation and melanin, making it attractive for quantitative skin chromophore mapping. Stimulated Raman scattering (SRS) in polarization‐maintaining fiber offers a compact, cost‐effective solution for multispectral excitation, producing discrete, red‐shifted wavelengths from a single pump source [9].

Here, we present a multispectral laser‐scanning photoacoustic microscopy (MS‐LS‐PAM) system that integrates the high spatial resolution of OR‐PAM, the speed and simplicity of LS‐PAM, and the functional imaging power of MS‐PAM for high‐resolution, label‐free imaging of skin chromophores, enabling accurate chromophore separation, mapping of saturated oxygen (sO_2_), and visualization of collagen and melanin distributions.

## 2 Materials and Methods

### 2.1 MS‐PAM System

**FIGURE 1:**
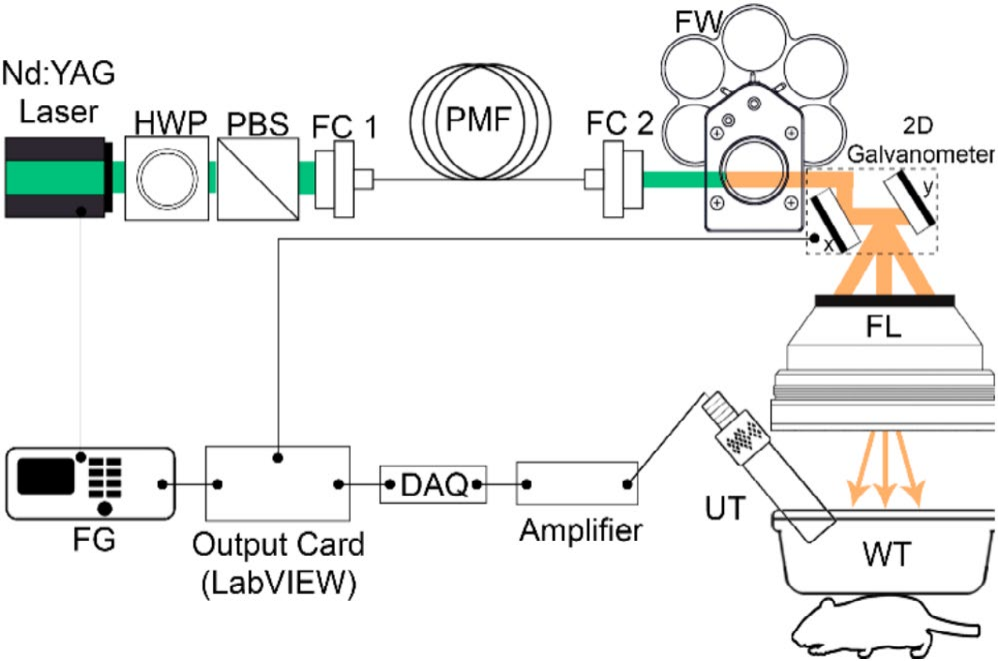
Schematic of the multispectral LS‐PAM system. FC1/FC2: Fiber couplers; FG: Function generator; FL: Focusing lens; FW: Filter wheel; HWP: Half‐wave plate; PBS: Polarizing beam splitter; PMF: Polarization maintaining fiber; UT: Ultrasound transducer; WT: Water tank.

The system configuration of the developed LS‐PAM system is shown in Figure 1. A frequency‐doubled Nd:YAG DPSS laser (RFH Tech Co. Ltd., S9 Series, China) operating at 532 nm, 13 ns pulse width, 5 kHz pulse repetition rate (PRR), with 20 μJ pulse energy (~100 mW average power) was used as the optical excitation source. To generate additional wavelengths from the 532 nm input, the SRS effect is employed in a 50 m polarization‐maintaining single‐mode fiber (PM‐SMF), as described in [3]. Briefly, a combination of a half‐wave plate (WPH10E‐532, Thorlabs, Newton, USA) and a polarizing beam splitter plate (PBS251, Thorlabs, Newton, USA) is used to divide the input beam into s‐ and p‐polarized components. The p‐polarized beam is launched into a PM‐SMF (HB450‐SC, Fibercore, United Kingdom), where nonlinear inelastic interactions generate SRS peaks with approximately 13 nm spacing. At the output of the PM‐SMF, a manually rotatable filter wheel (CFW6, Thorlabs, Newton, USA) is used to select specific wavelengths. Narrowband optical filters (10 nm bandwidth) centered at 532 nm (FLH532‐10, Thorlabs, Newton, USA), 545 nm (FBH545‐10, Thorlabs, Newton, USA), 558 nm (FBH558‐10, Thorlabs, Newton, USA) and 571 nm (FBH571‐10, Thorlabs, Newton, USA) are used to isolate the desired wavelengths. The input laser energy was chosen such that the output energies at 532, 545, and 558 nm are comparable. Output energy at each wavelength was measured using a photodetector (PDA36A2, Thorlabs, Newton, USA), and the pulse‐to‐pulse energy fluctuation was compensated using a reference photodiode (DET10A2, Thorlabs, Newton, USA). The selected laser beam is then directed onto a two‐axis galvanometer scanner (GVS202‐2D, Thorlabs, Newton, USA), which performs raster scanning of the beam across the sample. The scanning signals are generated using triangular waveforms in LabVIEW and transmitted to the galvanometer via an analog output interface (BNC 2110, National Instruments, USA). The scanned beam is focused using an F‐theta lens (FTH100‐1064, Thorlabs, Newton, USA) with a 10 cm focal length, providing a uniform spot across a 6 × 6 mm^2^ field of view. This lens has a numerical aperture of 0.06 and a spot size of 16 μm. A transparent 4 × 4‐in. water tank with a thin Saran wrap window is used for optical and acoustic coupling. Ultrasound gel is applied beneath the Saran wrap to ensure proper acoustic transmission to the target. A total of 10 000 points (100 × 100) were scanned in each frame. Pixel dwell time was 200 μs, yielding a total acquisition time of 2 s per frame. The configuration of the detection unit and associated electronics is described in detail in [10]. In related work, we have shown that integrating a miniaturized low‐noise preamplifier directly into the ultrasound transducer housing further increases PA signal amplitude and simplifies cabling in laser‐scanning PAM systems [11]. The present implementation uses an external amplifier chain, but the MS‐LS‐PAM architecture is fully compatible with such integrated detection units.

### 2.2 Characterization of the SRS Effect

SRS in optical fibers is strongly influenced by both the pump laser characteristics and the fiber properties. Among the laser parameters, pulse width, pulse energy, polarization, and PRR are the most critical [12]. In our implementation, the pulse width (13 ns) was fixed to maintain consistent peak power and avoid competing nonlinearities. Pulse energy directly determined the available photon density for Raman gain, while polarization was preserved in the p‐polarized state to maximize the polarization‐sensitive SRS process. PRR was varied across a wide range (5–50 kHz) to study its role in balancing signal strength with potential thermal and cumulative nonlinear effects. On the fiber side, the Raman gain coefficient, mode confinement, and effective interaction length govern SRS efficiency [13]. We employed PM‐SMF to preserve polarization over long distances, thereby enhancing Raman gain. Five fiber lengths (10, 20, 30, 40, and 50 m) were tested to examine the influence of propagation length on SRS onset and conversion efficiency. All spectra shown in results were measured at the fiber output after PM‐SMF and after applying the corresponding bandpass filters. The input pulse energy was increased systematically up to 40% of the fiber's measured damage threshold to ensure stable, repeatable operation. The minimum peak power required for SRS generation is given by (Equation 1):

(1) $$P_{\mathrm{th}}=\left(16.\;A_{\mathrm{eff}}\right)/\left(k_{p}.L_{\mathrm{eff}}.g_{R}\right)$$

where 𝑔_𝑅_ is the Raman gain coefficient, 𝑘_𝑝_ (0.5 ≤ 𝑘_𝑝_ ≤ 1) is the polarization factor, 𝐿_𝑒𝑓𝑓_ is the effective fiber length, and 𝐴_𝑒𝑓𝑓_ is the effective mode area [14]. Here, the factor 16 is dimensionless and arises from the analytic approximation of Raman threshold in single‐mode fiber. Although the exact Raman threshold depends on the coupled pump–Stokes equations, the closed‐form analytic approximation used here follows the standard derivation for SRS in single‐mode fiber, where the Stokes wave is assumed to grow from spontaneous Raman scattering and pump depletion is negligible. Under these assumptions, integrating the coupled equations yields an exponential growth condition for the Stokes field, and the threshold occurs when the Stokes power at the fiber output equals the pump power. Solving this condition produces a well‐known relation $P_{\mathrm{th}}\approx 16A_{\mathrm{eff}}/\left(k_{p}\mathrm{gR}L_{\mathrm{eff}}\right)$. This expression has been extensively validated in nonlinear fiber optics and is consistent with the derivation reported in Raman Gain Characterization in Standard Single‐Mode Optical Fibers. All variables have standard SI units: $A_{\mathrm{eff}}$ ($m^{2}$), $g_{R}$ ($\frac{m}{w}$), $L_{\mathrm{eff}}$ (m), $P_{\mathrm{th}}$ (W). This relation highlights that the SRS threshold can be tuned by adjusting fiber geometry, length, and polarization alignment. In this study, we investigated how input energy, fiber length, and PRR jointly determine the onset and strength of the SRS signal in PM‐SMF, under fixed pulse width and polarization conditions.

### 2.3 Animal Experiments

We performed two sets of in vivo experiments: one on the abdominal skin of a 3‐week‐old C57BL/6J (B6) mouse and the other on the abdominal skin of a 5‐week‐old SKH1 hairless mouse. The choice of these two strains allowed us to compare dermis with negligible melanin (nude SKH1) versus pigmented dermis containing measurable epidermal melanin (B6). This pairing provides both a baseline collagen‐dominant case and a physiologically relevant pigmented case. For both experiments, mice were anesthetized with 1%–2% isoflurane in oxygen, and body temperature was maintained with a heating pad. Residual hair was removed using a trimmer followed by a depilatory lotion, and the skin was cleaned with sterile saline. A thin layer of ultrasound gel was applied for acoustic coupling, and the mouse was positioned on the PAM stage with the abdominal skin gently secured to minimize motion. A 6 × 6 mm^2^ rectangular region of interest (matching the 100 × 100 scan grid) was selected. Following imaging, the mouse was humanely euthanized in accordance with AVMA guidelines. All procedures were approved by the Institutional Animal Care and Use Committee (IACUC) at the University of Illinois Chicago.

### 2.4 Signal Processing and Image Reconstruction

After acquiring a single raster‐scanned frame A‐lines from the MS‐LS‐PAM system (composed of multiple laser pulses, not a single‐pulse exposure), the raw PA signals were processed using an in‐house deep‐learning enhancement model developed specifically for this study (no pretrained networks were used) [15, 16]. Let $x\in {\mathbb{R}}^{4096}$ denote the raw input waveform. The encoder consisted of a sequence of fully connected layers with ReLU activations (4096 → 3072 → 1024 → 512 → 256), progressively suppressing noise and extracting salient temporal–spectral features. The resulting 256‐dimensional embedding was passed through a single‐head self‐attention mechanism with positional embeddings [17], as shown in (Equation 2):

(2) $$u=\tanh\left(W_{a}z\right),\alpha =\text{softmax}\left(W_{s}u\right),c=\alpha ⊙z$$

Producing an attention‐weighted latent representation.

A mirrored decoder (256 → 512 → 1024 → 3072) reconstructed a high‐SNR waveform $\hat{x}$. The network was trained end‐to‐end using paired datasets of single‐shot A‐lines and reference high‐SNR waveforms generated from 150× averaging.

Training used the Adam optimizer (learning rate $1\times 10^{-4}$), batch size 32, and 120 epochs. The loss function combined L1 reconstruction loss with a multi‐scale STFT loss, as defined in (Equation 3):

(3) $$L=\lambda_{1}{\left|\left|\hat{x}-x_{\mathrm{ref}}\right|\right|}_{1}+\lambda_{2}{\left|\left|\text{STFT}\left(\hat{x}\right)-\text{STFT}\left(x_{\mathrm{ref}}\right)\right|\right|}_{1}$$

With $\lambda_{1}=0.6$ and $\lambda_{2}=0.4$.

Data augmentation (Gaussian noise, amplitude jitter, temporal shifts) improved robustness and generalization.

Next, each time‐domain signal is converted to its envelope using a Hilbert transform [18]. Under linear thermoelastic conditions, the initial photoacoustic pressure and the total acoustic energy generated in the absorber are both proportional to the locally absorbed optical energy. In practice, the detected PA waveform represents the convolution of the true PA pressure with the impulse response of the ultrasonic detector, so any single‐point metric such as peak amplitude reflects both absorption and detector characteristics. To obtain a more robust and wavelength‐consistent metric, we use the integrated Hilbert envelope (AUC) of the PA signal as a surrogate for absorbed optical energy. Assuming a linear PA regime, wavelength‐independent detector response, and identical acquisition settings across wavelengths, the multiplicative effect of the detector response remains constant and is removed through the energy‐normalization step. Consequently, the AUC provides higher SNR than peak amplitude and offers a more stable and wavelength‐consistent representation of absorbed optical energy for multispectral unmixing. The envelope is then integrated over time using the trapezoidal numerical method [19] to calculate the signal energy. This energy value represents the PA intensity at that spatial location and is assigned to the corresponding pixel in the 2D image matrix. To enable quantitative comparison across wavelengths, the reconstructed images were further compensated based on the averaged pulse energy of each excitation wavelength. This energy compensation accounts for the differences in laser output and ensures that the image intensities reflect true relative chromophore absorption rather than differences in excitation power. This procedure results in four energy‐compensated PA images corresponding to excitation wavelengths of 532, 545, 558, and 571 nm. These four spectral images were then spatially aligned using MATLAB's structure‐based image registration tools. Although the present datasets are modest in size, continuous MS‐LS‐PAM acquisitions can generate high‐throughput A‐line streams that become storage‐limited in large‐area or longitudinal studies. To address this, we have previously developed an adaptive run‐length encoded discrete cosine transform (AR‐DCT) compression scheme that enables high‐fidelity real‐time compression of PAM data in LabVIEW while preserving image details [20]. This compression framework is complementary to the deep‐learning‐based denoising used here and could be integrated into future high‐volume MS‐LS‐PAM implementations.

### 2.5 Spectral Unmixing and Chromophore Quantification

The spectral unmixing process was formulated as a constrained linear least squares problem to estimate chromophore concentrations from the multispectral PA signals.

The PA area under the curve (AUC) represents the integrated PA signal energy, which corresponds to the optical absorption strength of the underlying chromophores. The AUC for each pixel was calculated as shown in (Equation 4):

(4) $$\mathrm{AUC}=\int_{t_{0}}^{t_{1}}|H\left\{p\left(t\right)\right\}\mathrm{dt}$$

where $p\left(t\right)$ is the time domain PA signal after background subtraction, and $H\left\{p\left(t\right)\right\}$ denotes the analytic signal obtained via the Hilbert transform. In practice, this integral was numerically computed in MATLAB using the trapezoidal rule. The operation integrates the absolute value of the analytic PA envelope over time, yielding a scalar AUC value proportional to the absorbed optical energy at that pixel and wavelength. The resulting AUC maps were subsequently energy‐normalized across wavelengths prior to spectral unmixing.

To perform unmixing, for each pixel the vector of normalized AUC values across wavelengths was arranged into a column vector b, and a matrix A was constructed using the known extinction coefficients of the chromophores. The system was solved by minimizing as in (Equation 5):

(5) $$\min_{x}{\left|\left|\mathrm{Ax}-b\right|\right|}^{2}\;\text{subject to}\;x\geq 0$$

where x contains the estimated concentrations of either HbO_2_ and Hb or collagen and melanin, depending on the pixel's location relative to the mask.

We solved each per‐pixel unmixing as a non‐negative least squares (NNLS) problem rather than unconstrained least squares via the pseudoinverse. The pseudoinverse yields minimum‐norm residuals but can produce negative concentrations and amplifies measurement noise along directions associated with small singular values (especially when the spectra matrix is ill‐conditioned) [21]. In our case, unmixing was split into two independent two‐parameter problems, HbO_2_/Hb inside vessel masks and collagen/melanin in surrounding dermis, each with known molar extinction coefficients and low condition numbers (7.5 for Hb and HbO_2_ and 41 for collagen and melanin), so numerical instability was not the limiting factor. Our primary requirement was physical plausibility: non‐negative concentrations for every pixel, which NNLS enforces. Using NNLS avoids the truncation‐induced bias that would arise from clipping negative unconstrained estimates and keeps derived metrics such as sO_2_ and the chromophore maps physically interpretable without subsequent adjustments. For these reasons, we used NNLS for all reported analyses [21]. A recent systematic evaluation of multispectral PAM decomposition algorithms likewise showed that physically constrained inversions (e.g., enforcing non‐negativity and regularization) are more robust to noise and spectral overlap than unconstrained least‐squares approaches [1]. Our NNLS + Tikhonov formulation follows these conclusions by explicitly incorporating both constraints and prior spectral information.

To improve stability under noise and reduce crosstalk between chromophores, the NNLS problem was reformulated with a.

Tikhonov regularization term in (Equation 6) [22, 23, 24]:

(6) $$\hat{c}\left(\lambda \right)=\arg\min_{c\geq 0}\;\left({\left|\left|\mathrm{Ac}-b\right|\right|}_{2}^{2}+\lambda^{2}{\left|\left|\mathrm{Lc}\right|\right|}_{2}^{2}\right)$$

where L is the regularization operator, and λ is the regularization parameter. The parameter λ was selected automatically using the L‐curve criterion, balancing the residual norm ${\left|\left|Ac-b\right|\right|}_{2}^{2}$ against the solution norm ${\left|\left|\mathrm{Lc}\right|\right|}_{2}$. The corner of the L‐curve (maximum curvature point) was taken as the optimal λ. This regularized NNLS stabilized solutions in regions of high spectral correlation and preserved non‐negativity, yielding smoother and more physically consistent chromophore maps. After estimating the chromophore concentrations, functional maps were calculated. sO_2_ was computed for vascular pixels using (Equation 7):

(7) $$sO_{2}=\frac{\left[\mathrm{Hb}O_{2}\right]}{\left[\mathrm{Hb}O_{2}+\mathrm{Hb}\right]}\times 100$$

For non‐vascular tissue, relative concentration maps of collagen and melanin were calculated from (Equations 8 and 9):

(8) $${\text{Collagen}}_{\text{relative}}=\frac{\left[\text{Collagen}\right]}{\left[\text{Melanin}+\text{Collagen}\right]}\times 100$$

(9) $$\text{Melani}n_{\text{relative}}=\frac{\left[\text{Melanin}\right]}{\left[\text{Melanin}+\text{Collagen}\right]}\times 100$$

These values represent the proportional contributions of each absorber, independent of absolute concentration.

**FIGURE 2:**
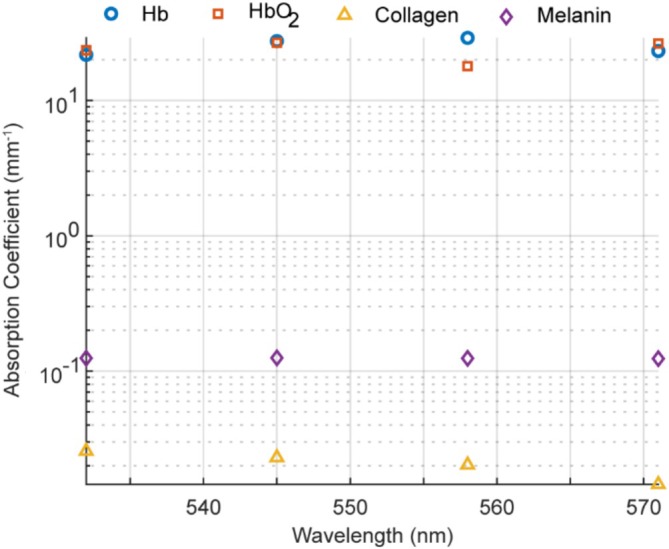
Absorption coefficients of Hb, HbO_2_, collagen, and melanin across the selected wavelengths.

The absorption coefficients in Figure 2 highlight the large contrast between blood chromophores (Hb and HbO_2_) and dermal chromophores (collagen and melanin) across our wavelength range. At all four wavelengths, Hb and HbO_2_ exhibit absorption values ranging from ~10–29 mm^−1^, which are three orders of magnitude higher than collagen (0.012–0.026 mm^−1^). Melanin, while stronger than collagen in absorption (0.12–0.13 mm^−1^), remains more than an order of magnitude lower than blood in this band. This pronounced separation in spectral magnitude justifies our approach of segmenting the analysis into vascular and non‐vascular regions: within the vessel mask, unmixing focuses solely on HbO_2_ and Hb, avoiding melanin/collagen interference; outside the mask, unmixing is restricted to collagen and melanin, where blood absorption is negligible [25] This targeted grouping improves quantitative stability and ensures physiologically meaningful concentration maps.

**FIGURE 3:**
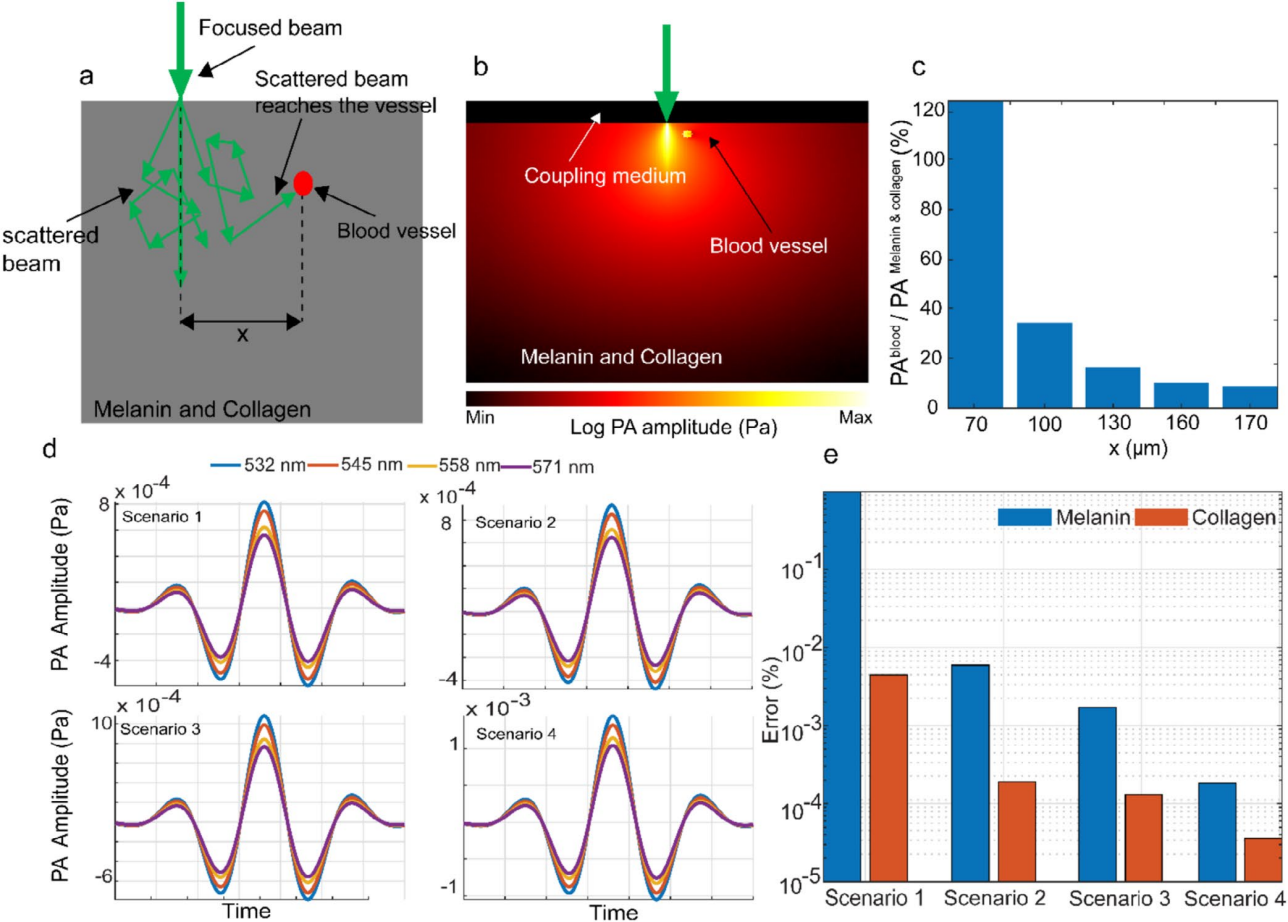
Photoacoustic simulation (a) MCX simulation schematic showing focused beam illumination of a melanin−/collagen‐rich region adjacent to a blood vessel and photon scattering paths. (b) Simulated PA amplitude distribution at 532 nm. (c) Ratio of vessel‐generated PA pressure to melanin/collagen PA pressure. (d) k‐Wave‐simulated PA waveforms at four wavelengths for four synthetic melanin–collagen mixtures: Scenario 1 (1%, 99%), Scenario 2 (5%, 95%), Scenario 3 (10%, 90%), and Scenario 4 (20%, 80%). (e) Corresponding melanin and collagen estimation errors for each scenario in log scale.

The input to the unmixing algorithm consisted of data collected at the four wavelengths. To isolate vascular structures where hemoglobin is predominantly present, we first generated a vessel mask using a Frangi filter [26] to the PA image at 545 nm (corresponding to an isosbestic point of HbO_2_ and Hb as it provided the best PA contrast with the vessels being most clearly visible), for vessel boundary detection. We applied a threshold of 0.1 to the Frangi filter to create the vessel mask. To prevent HbO_2_ and Hb PA signals from contaminating regions designated for melanin and collagen analysis, we conducted a Monte Carlo eXtreme (MCX) simulation [27]. The goal was to determine the minimum distance from a blood vessel at which vessel‐generated PA pressure drops below 10% of the maximum PA pressure from melanin or collagen when illuminating those regions. As shown in Figure 3a–c, the simulation schematic, PA distribution at 532 nm, and the computed PA ratio (blood vs. melanin/collagen) respectively, reveal that melanin‐ or collagen‐rich areas within 150 μm of a vessel receive more than 10% of their PA signal from the vessel (the value drops below 10% at 170 μm). This effect results from the substantially higher optical absorption of blood (e.g., at 532 nm: Hb = 21.7360 mm^−1^, HbO_2_ = 23.499 mm^−1^) compared with melanin (0.124678 mm^−1^) and collagen (0.025647 mm^−1^), combined with the absorption of scattered photons by adjacent vessels. To mitigate this effect, we expanded vessel boundaries in the binary mask by 160 μm, defining this buffer as a marginal safe area. In our unmixing workflow, hemoglobin components were estimated only inside the vessel mask, while melanin and collagen were quantified exclusively outside the expanded boundary. This spatial separation minimizes physiologically irrelevant spectral unmixing artifacts and enhances estimation specificity.

For validation, we generated synthetic regions with controlled collagen–melanin compositions of (99:1), (95:5), (90:10), and (80:20). The 99:1 mixture reflects physiological conditions in epidermis or dermis [28], with B6 mice having significantly more melanin. The higher melanin fractions (90:10, 80:20) were included as deliberate stress‐test scenarios, representing potential partial‐volume contamination or superficial epidermal melanin intrusion that can bias OR‐PAM measurements. This design ensures that unmixing performance is validated under both expected and elevated pigment conditions. The corresponding absorption and scattering coefficients at four wavelengths were taken from Figure 2 and Table 1. PA signal generation from these regions was simulated using the k‐Wave toolbox [31] on a 512 × 512 grid with 10 μm pixel spacing. A 5 mm flat transducer was modeled as an array of 500 omnidirectional point transducers, with spatial averaging to simulate directivity. The transducer had a 2.25 MHz center frequency and 66% bandwidth for recording PA signals.

**TABLE 1:** Scattering coefficient of collagen and melanin (mm^−1^).

WL (nm)	Collagen [29]	Melanin [30]
532	0.13834	0.022589
545	0.14249	0.022654
558	0.14414	0.021333
571	0.14509	0.019359

The speed of sound was assumed to be 1500 m/s. Figure 3d,e present the recorded PA waveforms at all four wavelengths for each composition ratio, along with the true and estimated melanin and collagen concentrations. The estimation error for each chromophore was computed pixel‐wise as the absolute percentage difference between the estimated and true concentrations using (Equation 10):

(10) $$\text{Error}\;\left(\%\right)=\frac{|C_{\text{true}}-C_{\mathrm{est}}|}{C_{\text{ture}}}\times 100$$

where $C_{\text{true}}$ and $C_{\mathrm{est}}$ are the ground truth and estimated concentrations, respectively. As shown, our proposed method estimates melanin and collagen concentrations with an error of less than 0.18%.

## 3 Results and Discussion

**FIGURE 4:**
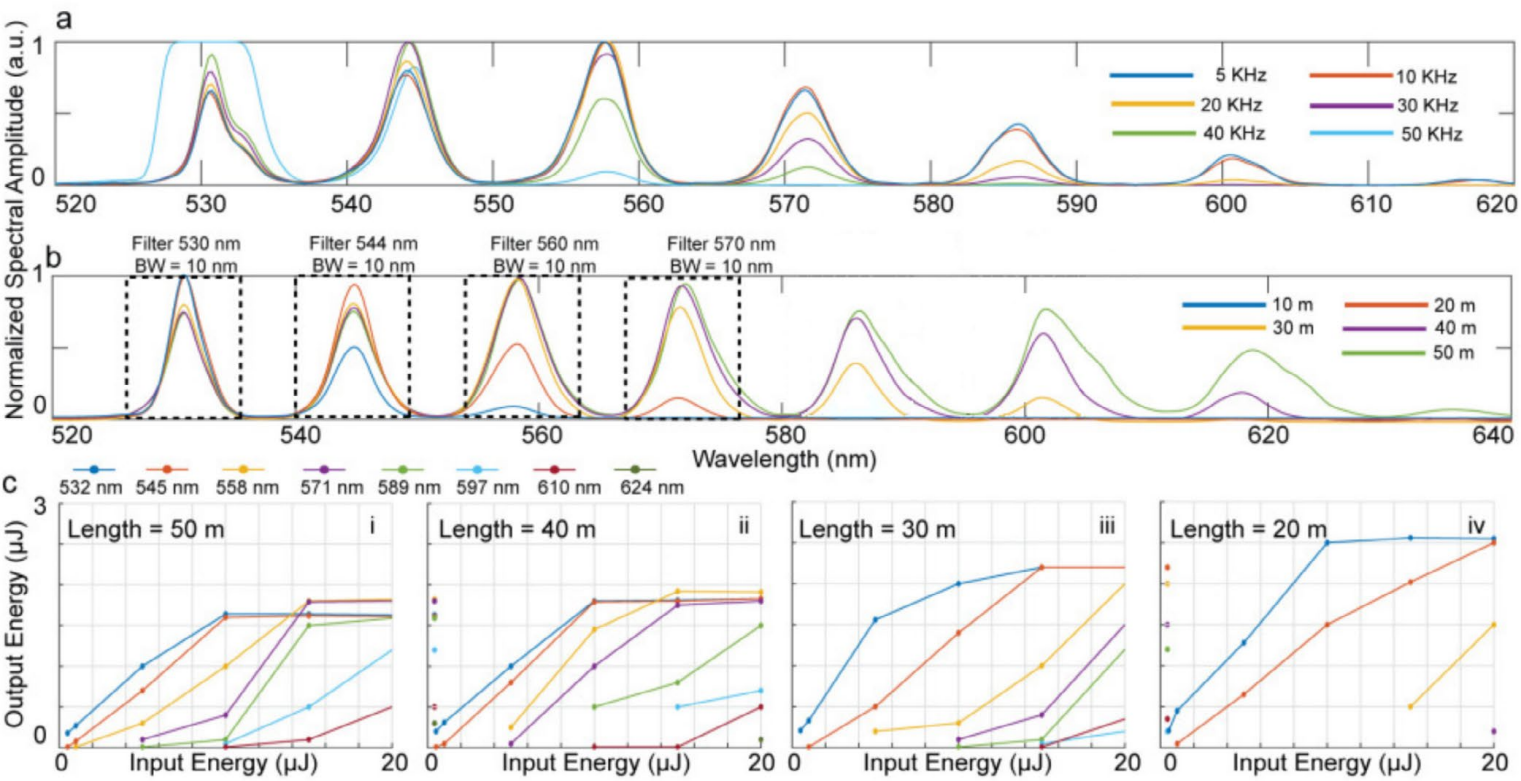
Combined characterization of SRS cascade generation. (a) Effect of pulse repetition rate (PRR) on normalized spectral amplitude for a fixed fiber length of 50 m and input pulse energy of 20 μJ. (b) Effect of fiber length (10 to 50 m) at fixed PRR (5 kHz) and input energy (20 μJ). Dash lines show the bandwidth (BW) for each of the filters used in this experiment. (c) Output energy versus input pulse energy for individual wavelengths at four fiber lengths (20, 30, 40, and 50 m). Although only the first four wavelengths were utilized in our experiments (boxes shown in (b)), information at the higher wavelengths is available via the SRS setup. Each curve color corresponds to a different input pulse energy.

We investigated the impact of PRR on the SRS cascade effect, using PRR values ranging from 5–50 kHz. For this experiment, the pulse width (13 ns), optical fiber length (50 m), and input energy (20 μJ) were held constant. As shown in Figure 4a, increasing the PRR led to a noticeable reduction in the strength and extent of the SRS cascade. At lower PRRs of 5 and 10 kHz, a full cascade of wavelengths was generated, spanning from 532 to 571 nm with ~13 nm spacing between peaks. In contrast, at 50 kHz, only two wavelengths 532 and 545 nm were observed, with the 532 nm peak exhibiting saturation due to the suppression of higher‐order Stokes generation. This attenuation in SRS efficiency at higher PRRs is attributed to insufficient time for the fiber to dissipate thermal energy between pulses and a corresponding reduction in peak power, which collectively diminishes the nonlinear interaction strength required for cascading Raman processes. We next examined the effect of fiber length on the generation of SRS cascaded wavelengths. Fiber lengths ranging from 10–50 m were tested, while keeping the laser parameters constant: pulse width of 13 ns, PRR of 5 kHz, and input energy of 20 μJ. The results, presented in Figure 4b, demonstrate a clear improvement in the SRS effect with increasing fiber length. Specifically, the 10 m fiber produced only two distinct wavelengths, 532 and 545 nm, whereas the 40 and 50 m fibers generated a complete cascade of Stokes‐shifted wavelengths from 532 nm up to 620 nm in ~13 nm increments. This progressive spectral broadening with longer fibers is attributed to the increased interaction length, which facilitates stronger nonlinear coupling and energy transfer between successive vibrational modes of the silica molecules in the fiber core.

We further investigated the influence of input pulse energy on the generated SRS spectrum across different optical fiber lengths, specifically 20, 30, 40, and 50 m. The corresponding results are summarized in Figure 4c. As the fiber length increased, the threshold energy required to initiate SRS decreased, and a more extensive cascade of Stokes‐shifted wavelengths was observed. This trend is attributed to the increased interaction length, which enhances the probability of nonlinear scattering events between pump photons and silica molecules within the fiber core. Conversely, in shorter fibers, while fewer Stokes components were generated, the individual spectral lines exhibited noticeably higher energies. This behavior arises from the reduced number of scattering events, which limits the redistribution of pump energy across multiple wavelengths. As a result, a greater fraction of the input energy is concentrated into fewer spectral components, leading to stronger individual SRS peaks. These findings underscore the trade‐off between spectral breadth and individual peak intensity as a function of fiber length and input energy.

**FIGURE 5:**
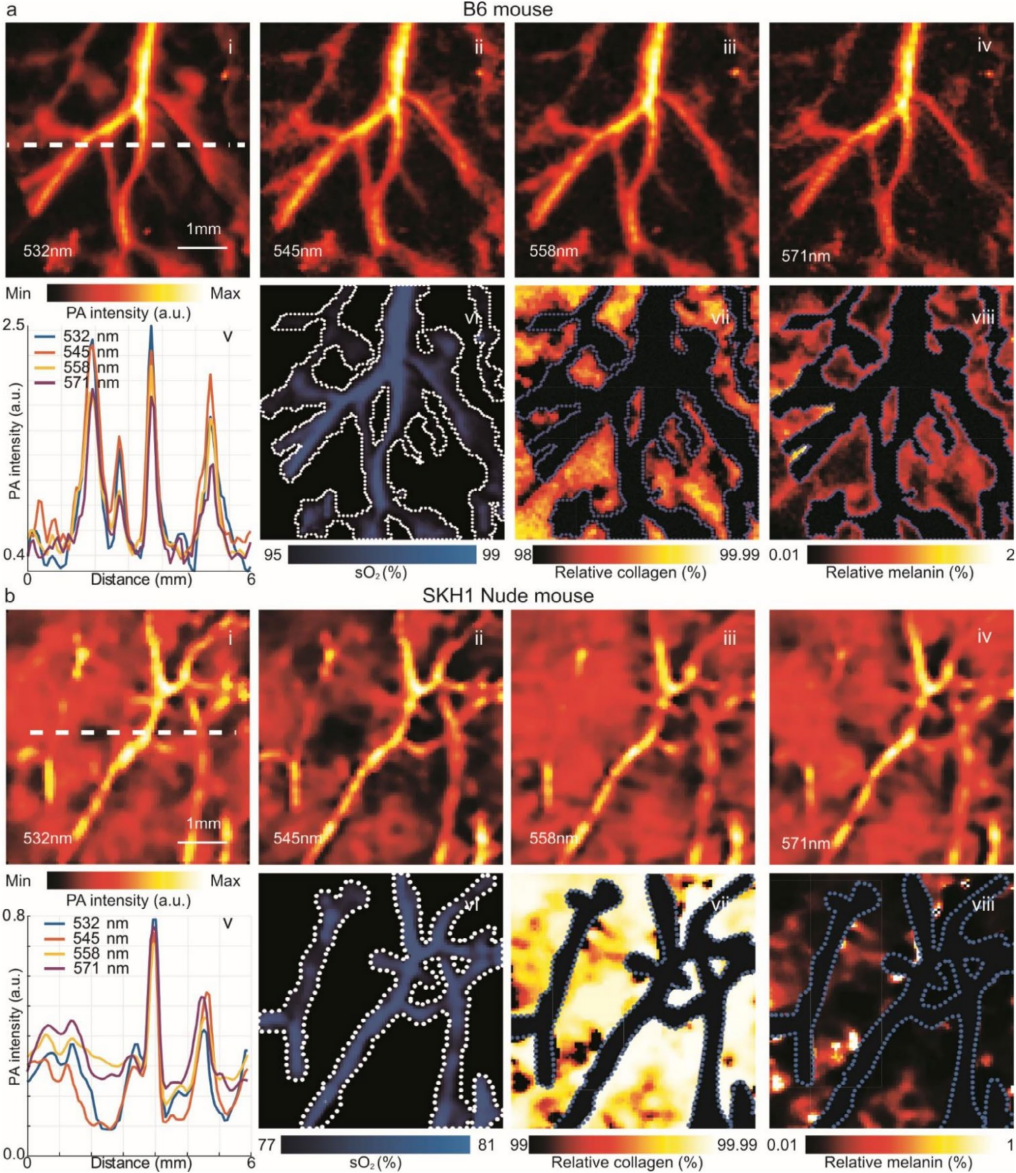
Results of photoacoustic images. (a) PA amplitude images of the B6 mouse skin at 532 nm (i), 545 nm (ii), 558 nm (iii), and 571 nm (iv). (v) Line profile of PA intensity along the dashed line in (i). (vi–viii) Unmixed maps showing vascular oxygen saturation (sO_2_), relative collagen, and relative melanin distributions for the B6 mouse. (b) Corresponding PA amplitude images of SKH1 hairless (nude) mouse skin at 532 nm (i), 545 nm (ii), 558 nm (iii), and 571 nm (iv). (v) Line profile of PA intensity along the dashed line in (i). (vi–viii) Unmixed maps showing vascular sO_2_, relative collagen, and relative melanin for the nude mouse. Note that collagen dominates dermal composition in both models, whereas melanin is sparse in nude skin (~0.1%) and higher in pigmented B6 skin (~2%), consistent with biochemical reports. Dash dot outlines indicate vessel boundaries excluded from analysis.

Multispectral imaging of mouse abdominal skin was conducted on both mice using the proposed MS‐LS‐PAM system. For the B6 mouse, as illustrated in Figure 5a(i–iv), the PA images at individual wavelengths reveal vascular networks with strong contrast and well‐defined boundaries. The variation in signal intensity across the four wavelengths reflects the differing absorption spectra of hemoglobin and other endogenous chromophores in pigmented skin. The line profile in Figure 5a(v) quantifies the PA signal along the white dashed line in Figure 5a(i), showing spectral differences between vessels and adjacent dermal tissue. Figure 5a(vi–viii) present the unmixed functional and structural maps derived from multispectral analysis. Figure 5a(vi) shows arterial and venous branches with distinct oxygenation distributions, consistent with normal microvascular physiology. Also, vessel segments with higher oxygen saturation are consistent with arterioles, whereas segments with lower oxygen saturation are characteristic of venules. Relative collagen and melanin maps were computed outside of the vessel mask to characterize dermal composition and range up to 2% melanin. In Figure 5a(vii), (viii), the dotted dashed line outlines mark vascular regions excluded from the unmixing analysis. The collagen map shows a dense, spatially continuous dermal distribution, consistent with collagen's role as the primary extracellular matrix protein in B6 mouse skin. The melanin map demonstrates sparse but clearly detectable signals localized to basal epidermal regions and perivascular sites, reflecting the pigmentation typical of B6 mice. In B6 mouse skin, melanin is present in lower concentrations than collagen. Takeuchi et al. reported that dorsal skin of B6 mice contains approximately 1390 ng/mg dry skin of melanin, composed entirely of eumelanin [32]. In contrast, collagen is the dominant structural component of dermis. Sayama et al. measured collagen content in B6 dorsal skin using the Sircol dye‐binding assay, reporting concentrations on a per‐extract‐volume basis [33]. When normalized to tissue mass using typical Sircol extraction ratios (1:10–1:20 w/v) and correcting for skin water content (~70%), these values correspond to roughly 22 000–45 000 ng/mg dry skin. Independent hydroxyproline‐based determinations of total collagen yield higher values, on the order of 525 μg/mg dry skin (≈525 000 ng/mg), consistent with collagen comprising the majority of skin dry weight. Taken together, these estimates indicate that collagen accounts for approximately 95%–99.5% of the collagen/melanin pool, whereas melanin contributes only 0.5%–5%. While controlled in vitro phantoms were not included in this study, the extracted collagen and melanin proportions closely match published biochemical measurements for B6 and SKH1 mouse skin, providing independent validation of the physiological plausibility of the estimated chromophore distributions. The lower end of this range reflects Sircol‐derived values (which measure only extractable collagen), while the upper end reflects hydroxyproline‐based total collagen content. Thus, even under conservative assumptions, collagen dominates the biochemical composition of B6 skin, with melanin representing only a minor fraction.

Noninvasive OCT studies of human skin report concordant structural and optical findings, including atlas‐level measurements of epidermal and dermal morphology on sun‐exposed areas, automated detection of skin layer boundaries, and quantitative scattering analyses [34, 35, 36]. Several groups have also developed hardware and software approaches to mitigate OCT artifacts—such as dynamic‐focus en‐face imaging, refractive‐index correction, and learnable despeckling frameworks—to improve visualization of clinically relevant microstructures [37, 38, 39, 40, 41]. Together, these OCT advances emphasize the importance of accurate modeling of skin microstructure and light propagation, and they provide a natural roadmap for future co‐registered OCT/PA systems that could jointly exploit structural and chromophore contrast in dermatologic applications. For the hairless mouse, as illustrated in Figure 5b(i–iv), the enhanced PA images at individual wavelengths reveal well‐defined vascular structures. The variation in signal intensity and vessel contrast across wavelengths corresponds to the distinct absorption characteristics of hemoglobin and other endogenous chromophores. The line profile in Figure 5b(v) quantifies the PA signal along the white dashed line indicated in Figure 5b(i), highlighting differences in spectral responses across vessels and surrounding tissue. Figure 5b(vi) illustrates the calculated sO_2_ map. Vessel segments exhibited heterogeneous oxygen saturation, with values of 77%–81%. Prior studies of murine microvasculature have reported higher oxygenation, with arterioles approaching ~99% and venules ~81% in the mouse ear [42], and human skin microvascular oxygenation spanning a broad physiological range across sites (e.g., ~65% in volar forearm to ~90% in fingertip) [43]. Our slightly lower values are consistent with the experimental context: imaging was performed at the end of an extended in vivo session, during which the animal was under prolonged anesthesia. Previous reports have documented that extended isoflurane exposure can depress microvascular oxygenation [44], and in our case the mouse was exhibiting signs of severe physiological stress at the conclusion of imaging. These factors likely contributed to the downward shift in measured sO_2_ relative to the higher ranges reported in the literature. Beyond normal skin, multispectral PA imaging has been applied broadly to interrogate pathologic changes in tissue oxygenation and microvascular function. Nasri et al. reviewed how PAI can quantify tumor hypoxia and hypoxia‐regulated signaling to study tumor progression and treatment response [45], while Benavides‐Lara et al. used PAM to monitor Piezo1‐dependent remodeling and vasoconstriction of resistance arterioles in a hypertensive mouse model [45]. Closely related MS‐LS‐PAM and high‐frequency PA/US systems have also been demonstrated for large‐field sO_2_ mapping and for in vivo assessment of acute skin injury, such as chemical burns [46, 47]. Relative collagen and melanin maps were computed outside the vascular mask regions to characterize dermal composition. As shown in Figure 5b(vii,viii), dotted dashed lines indicate vessel boundaries excluded from the analysis. The collagen map reveals a dense and spatially continuous distribution, with a mean relative abundance of 99.9%, while melanin appears sparsely distributed, averaging 0.1%. This pronounced collagen dominance is consistent with histological descriptions of SKH1 mouse dermis as being rich in extracellular matrix proteins, particularly collagen, which maintains dermal integrity despite the absence of hair follicles [48]. The low melanin levels are also expected, since SKH1 mice exhibit greatly reduced pigmentation compared with pigmented strains, with only localized deposits reported in basal epidermal layers or appendage remnants [28]. Furthermore, our observed collagen–melanin balance agrees with the qualitative trends reported by Hult et al. [49], who noted abundant collagen and sparse melanin in healthy dermis adjacent to melanoma lesions. Together, these literature reports support the physiological plausibility of our unmixing results. Although the system successfully resolved hemoglobin, melanin, and collagen, other endogenous absorbers such as lipids and water have optical absorption coefficients in the visible range that are orders of magnitude lower than those of the dominant chromophores at the investigated wavelengths (532–571 nm), lipid absorption ranges from 3 × 10^**−6**^–9 × 10^**−6**^ mm^**−1**^ and water absorption from 1 × 10^**−4**^ to 2 × 10^**−4**^ mm^**−1**^, typically producing PA signals on the order of 10^−5^ [50], making them undetectable without ultra‐low‐noise conditions and high dynamic range acquisition. To evaluate whether these weak absorbers could still be recovered, we performed numerical simulations including all six chromophores (Hb, HbO_2_, melanin, collagen, lipid, and water) using the same vessel‐masking strategy described in the paper. The condition number of the molar extinction coefficient matrix for the non‐blood absorbers (collagen, melanin, lipid, and water) was over 60 million, indicating severe ill‐conditioning. Values above ~1000 are generally considered problematic for stable inversion [51]. Simulations with 64‐bit precision (assuming that the noise equivalent pressure is smaller than the pressure equivalent to the least significant bit (LSB) of the analog to digital converter) yielded excellent recovery of all absorbers; however, when limited to the 16‐bit acquisition depth available in our experimental system, the unmixing accuracy for lipid and water degraded dramatically. Given this instability, and their negligible absorption in our wavelength range, lipid and water were excluded from the experimental unmixing to ensure quantitative robustness of the reported results.

One potential solution is to use longer excitation wavelengths, such as 1064 nm and beyond, where lipid absorption is significantly higher and PA imaging can distinguish lipid‐rich regions even in the presence of blood and other tissue [52, 53]. For example, Lv et al. recently used dynamic synthetic‐scanning PAT at 1064 nm, combined with Monte‐Carlo‐based fluence correction, to track hepatic and renal clearance pathways of exogenous probes in vivo, highlighting the potential of long‐wavelength PA imaging for deep, quantitative whole‐organ functional studies [54]. Melanin remains the dominant absorber across the 1064–1275 nm range, the wavelength range our fiber can produce [55], with absorption coefficients between 3.21 and 5.52 mm^−1^, whereas Lipid absorption increases in the 1175–1225 nm region, reaching approximately 0.086 mm^−1^ at 1225 nm [38], with another local maximum near 1175 nm [56]. Water absorption follows a similar trend to lipids, reaching 0.10225 mm^−1^ at 1175 nm, while collagen remains the weakest absorber but within range of the others (0.03186 mm^−1^ at 1175 nm). To assess the feasibility of separating these components, we calculated the condition number of the molar extinction coefficient matrix for the non‐blood chromophores (melanin, collagen, lipid, and water) over this wavelength range. It exceeded 11 000, which is better than 532 nm, but still highly ill‐conditioned, so that even modest noise or quantization errors would be strongly amplified during unmixing, making accurate recovery of lipid and water challenging in a standard in vivo setting. Numerical simulations confirmed that although with 64‐bit precision, all six absorbers could be separated successfully, the 16‐bit acquisition depth of our current system, would yield an error of more than 90%. For this reason, we realized that even if we had switched to the higher wavelength, we would not be able to effectively spectrally isolate lipid and water by SRS. Therefore, their analysis was reluctantly excluded. Nonetheless, longer wavelengths remain a promising option for future LS‐PAM systems optimized for lipid‐rich tissue imaging, where increased absorption at these wavelengths can be exploited with higher dynamic range detection and improved SNR. A limitation of our spectral unmixing paradigm is that we assumed that photoacoustic AUC scales with molar absorption for all chromophores. This is not strictly correct, due to the Grüneisen coefficient, that is, the relative efficiency with which absorbed energy is converted into acoustic waves, can differ among chromophores and tissue compartments [57]. However, the pairwise comparison of Hb and HbO_2_, both of similar composition and both contained within red blood cells, will have similar Gruneisen coefficients. Collagen and melanin, both polymeric in nature, may as well. However, collagen's well‐ordered fibrillar ECM localization could result in a higher Grüneisen coefficient than amorphous, granular melanin. In this case, we may have overestimated collagen content relative to melanin, especially in the B6 mouse model.

Compared to supercontinuum lasers, such as those from NKT or Fianium, our SRS‐based fiber source provides higher pulse energies (typically in the μJ range) at kHz repetition rates, making it more effective for PAM, where signal strength and spectral specificity are critical. Supercontinuum sources deliver ultra‐broadband output (e.g., 450–2400 nm) with excellent tunability, but they generally emit low energy per nm (~pJ) due to their high (100 kHz–MHz) repetition rates, which limits photoacoustic signal strength unless additional averaging or filtering is applied [58]. In contrast, SRS systems generate a small set of discrete, high‐energy wavelengths (e.g., 545 and 558 nm), spaced by the Raman shift of silica fiber, which align well with the absorption peaks of skin chromophores and therefore provide efficient multispectral excitation in a compact and cost‐effective format [12, 13].

In this study, we selected 532 nm as the pump wavelength rather than 1064 nm. While 1064 nm offers deeper penetration and stronger lipid absorption, hemoglobin absorption at this wavelength is very weak, making it unsuitable for high‐contrast microvascular imaging. By contrast, 532 nm lies within the strong absorption band of both Hb and HbO_2_, providing superior sensitivity for vascular oxygenation mapping [42]. In addition, shorter visible wavelengths enable tighter optical focusing, which improves lateral resolution in optical‐resolution PAM [59]. A practical advantage is that 532 nm also serves as an efficient pump for generating cascaded visible Raman lines (545–571 nm) via stimulated Raman scattering, which overlap with absorption peaks of hemoglobin, melanin, and collagen [60]. For these reasons, vascular contrast, resolution, and spectral coverage, we based our system on 532 nm excitation. Looking forward, combining both 532 and 1064 nm excitation would provide complementary advantages. While 532 nm is optimal for resolving microvasculature and pigment distributions in superficial skin, 1064 nm penetrates more deeply into tissue and exhibits stronger absorption by lipids and water [52, 53]. Dual‐wavelength excitation would therefore enable simultaneous assessment of vascular function, dermal collagen/melanin distribution, and deeper lipid‐ or water‐rich structures in a single system. Such a configuration would broaden the diagnostic capability of LS‐PAM and support applications ranging from cutaneous microcirculation mapping to metabolic and lipid‐related disease assessment. While our method enables label‐free functional and structural imaging relevant to dermatology and diagnostics, limitations include its restricted visible‐range sensitivity, dependence on accurate calibration and vessel masking, and reliance on deep learning enhancement, which may be dataset‐dependent.

Note that collagen dominates dermal composition in both models, whereas melanin is sparse in nude skin (~0.1%) and higher in pigmented B6 skin (~2%), consistent with biochemical reports. Dashed dot lines outlines indicate vessel boundaries excluded from analysis.

Together, these experiments demonstrate that collagen dominates dermal composition in both strains, but with markedly different melanin contributions: ~0.1% in SKH1 hairless versus 0.5%–5% in C57BL/6J. This consistency with reported biochemical ranges supports the physiological plausibility of our unmixing results.

## 4 Conclusion

The proposed LS‐PAM system with spectral capability centered at 532 nm enables high‐resolution, label‐free imaging of skin microvasculature and dermal structure. The choice of 532 nm as the pump wavelength allows efficient generation of tunable visible outputs via SRS in the fiber, and subsequent spectral filtering yields discrete wavelengths well aligned with the absorption peaks of hemoglobin species, melanin, and collagen. This cost‐efficient, fiber‐based configuration reduces system complexity while maintaining spectral flexibility for multi‐chromophore imaging. Deep learning was used to enhance signal quality, a process we anticipate will become ever more commonplace in the near future. A key innovation of this work is the targeted spectral unmixing strategy in which blood chromophores (HbO_2_ and Hb) are processed separately from dermal pigments (collagen and melanin), leveraging their distinct spectral profiles to improve unmixing accuracy. This is paired with an NNLS unmixing algorithm that enforces physical non‐negativity. Applied to in vivo mouse skin, the system produced sO_2_ maps of vessel segments ranging from 77%–81% in SKH1 hairless mice, consistent with reported venular oxygenation in murine cutaneous microvasculature. In these melanin‐deficient mice, collagen maps revealed dense, continuous distributions with a mean relative concentration of 99.99%, while melanin signals were negligible (~0.01%). In contrast, C57BL/6J (B6) pigmented mice exhibited collagen as the dominant component (98%–99.5%) with melanin contributing a physiologically minor but detectable fraction (0.5%–2%), localized primarily to basal epidermal regions and appendage‐associated sites. These relative proportions are in line with biochemical and histological reports, confirming that the unmixing strategy recovers physiologically meaningful chromophore distributions across distinct pigmentation contexts. Together, these results demonstrate that the LS‐PAM system provides both functional (oxygenation) and compositional (collagen/melanin) information in a single imaging session with high spatial resolution and physiological plausibility. Future work will expand the accessible wavelength range to target additional chromophores, implement real‐time reconstruction for clinical translation, and conduct longitudinal imaging studies to track skin disease progression and therapeutic response.

## Funding

This work was supported by the National Institutes of Health R01EB027769‐01, R01EB02866101.

## Conflicts of Interest

The authors declare no conflicts of interest.

## Data Availability

The data that support the findings of this study are available from the corresponding author upon reasonable request.
